# Variability and sex-dependence of hypothermic neuroprotection in a rat model of neonatal hypoxic–ischaemic brain injury: a single laboratory meta-analysis

**DOI:** 10.1038/s41598-020-67532-2

**Published:** 2020-07-02

**Authors:** Thomas R. Wood, Julia K. Gundersen, Mari Falck, Elke Maes, Damjan Osredkar, Else Marit Løberg, Hemmen Sabir, Lars Walløe, Marianne Thoresen

**Affiliations:** 1 Department of Physiology, Institute of Basic Medical Sciences, University of Oslo, Domus Medica, Sognsvannsveien 9, 0372 Oslo, Norway; 2 Department of Pathology, Oslo University Hospitals and University of Oslo, Oslo, Norway; 3 Neonatal Neuroscience, School of Clinical Sciences, University of Bristol, Bristol, UK; 4 Present Address: Department of Pediatrics, University of Washington Medical School, Seattle, USA; 5 Present Address: Department of Pediatric Neurology, University Children’s Hospital, Ljubljana, Slovenia; 6 Present Address: Department of Neonatology and Pediatric Intensive Care, Children’s Hospital University of Bonn, Bonn, Germany

**Keywords:** Diseases of the nervous system, Encephalopathy, Brain injuries, Paediatric research, Preclinical research

## Abstract

Therapeutic hypothermia (HT) is standard care for term infants with hypoxic–ischaemic (HI) encephalopathy. However, the efficacy of HT in preclinical models, such as the Vannucci model of unilateral HI in the newborn rat, is often greater than that reported from clinical trials. Here, we report a meta-analysis of data from every experiment in a single laboratory, including pilot data, examining the effect of HT in the Vannucci model. Across 21 experiments using 106 litters, median (95% CI) hemispheric area loss was 50.1% (46.0–51.9%; n = 305) in the normothermia group, and 41.3% (35.1–44.9%; n = 317) in the HT group, with a bimodal injury distribution. Median neuroprotection by HT was 17.6% (6.8–28.3%), including in severe injury, but was highly-variable across experiments. Neuroprotection was significant in females (*p* < 0.001), with a non-significant benefit in males (*p* = 0.07). Animals representing the median injury in each group within each litter (n = 277, 44.5%) were also analysed using formal neuropathology, which showed neuroprotection by HT throughout the brain, particularly in females. Our results suggest an inherent variability and sex-dependence of the neuroprotective response to HT, with the majority of studies in the Vannucci model vastly underpowered to detect true treatment effects due to the distribution of injury.

## Introduction

The use of animal research has dramatically advanced human knowledge of physiology, pathology, and science-based medicine^1^. However, despite centuries of improvement in the methodological and ethical approaches to experiments involving animals^1^, preclinical research across all fields of medicine has also significantly underperformed with regards to the translation of robust treatments for complex diseases in humans^2^.

For perinatal asphyxia and subsequent hypoxic–ischaemic encephalopathy (HIE), just one treatment so far, therapeutic hypothermia (HT), has emerged from the preclinical research to provide a robust treatment effect in term newborns with moderate-to-severe neurological injury^3^. A recent meta-analysis of those clinical trials found that the numbers needed to treat (NNT) were 11 to reduce mortality, and 8 to reduce major neurodevelopmental disability^3^. However, HT is not universally neuroprotective. Meta-analyses of the original large clinical trials of HT found that 40–50% of treated infants still experienced a poor outcome (death or severe disability)^4^, though more recent trials suggest it is now around 30%^5^.

In the most widely-researched model of neonatal hypoxic–ischaemic (HI) brain injury, the Vannucci model of unilateral HI, our group and others have traditionally found that HT provides around 40% reduction in neuropathology score and tissue loss^6–9^. This neuroprotective effect of HT was corroborated in larger animal models^10–12^, leading to incremental pilot studies in term newborn asphyxiated neonates, which formed the basis of large clinical trials^13–18^. The use of HT for infants with moderate-to-severe HIE can therefore be acknowledged as one of the few success stories of translational research for neurological diseases. Despite this, the neuroprotective effect of HT in preclinical studies remains generally greater than that seen clinically, and a number of possible explanations for this gap have been postulated. This includes the generally heterogeneous clinical population compared to highly-standardised animal models, as well as the fact that HT may not be beneficial after severe HI brain injury, or in the setting of certain types of systemic inflammation^6,19–21^. It is also generally accepted that published preclinical studies are more likely to show positive results than negative, and the low power of studies using small treatment groups is likely to exaggerate treatment benefits^22^.

Here, we aim to address some of these problems by collating and analysing all experiments in our standardised Vannucci rat model of unilateral HI brain injury in postnatal-day seven (P7) rats. The data spans a 3-year period employing the standard P7 Vannucci model in our laboratory. It includes all pilot data, as well as results from published studies, where an HT treatment group was included. The aim is to gain a greater insight into the usefulness and limitations of the model itself, as well as the effects of HT, and discuss a framework for future work investigating neonatal HIE in animal models.

## Methods

### Study design

From January 2013 to February 2016, experiments examining the effects of HT were performed 21 times in our laboratory as part of various experimental treatment protocols. All successfully-completed experiments that included our “standard” moderate HI injury (described below), a control normothermia (NT) group and HT group, were included in the analysis. Data from a total of 106 litters with 1,042 pups was available from 21 experiments that had both an NT control group and standard HT treatment group (Table 1). Results from one set of five consecutive experiments (experiment numbers 7–11, n = 126 animals; NT n = 62; HT n = 64) were previously published^6^. All other experiments (numbers 1–6 and 12–21) were previously unpublished. Ten of the 21 experiments included one or more other treatment groups in addition to the standard NT and HT groups as part of a number of pilot experiments. Animals from these groups were not included in the analysis. A total of 420 pups were excluded from this analysis. This included 51 pups that died during ligation (n = 30), hypoxia (n = 12), or the survival period (n = 9). An additional 369 pups were excluded because they carried temperature probes during HI and treatment (n = 108), or due to being in treatment groups other than standard NT and HT (n = 261). The final number of included animals was n = 622 (n = 329 females, n = 293 males). Survival was always 1 week. Two-to-four of the same five experienced investigators (TRW, MF, EM, DO, and HS) performed the surgical and experimental procedures in every experiment.

**Table 1:** Severity of injury and hypothermic neuroprotection.

Exp	Date	Median percent area loss (95% CI)				Median difference	% Neuroprotection	Barometric pressure (mmHg)
		NT	(N =)	HT	(N =)			
1	10/01/2013	42.4 (28.2–51.4)	16	44.4 (27.2–49.9)	14	− 2.0	− 4.7	761
2	08/02/2013	29.8 (5.5–43.1)	14	19.8 (2.5–26.9)	14	10.0	33.5	763.5
3	18/03/2014	33.7 (9.8–50.5)	11	47.6 (37.1–52.0)	13	− 13.9	− 41.2	748.5
4	25/04/2014	10.6 (1.9–21.5)	15	20.3 (2.3–22.9)	15	− 9.7	− 92.4	769.6
5	15/05/2014	48.6 (26.4–58.1)	11	27.9 (5.5–41.0)	13	20.7	42.7	773.2
6	13/06/2014	45.2 (25.9–56.4)	13	54.8 (36.7–58.7)	14	− 9.6	− 21.3	760.7
7	14/08/2014	58.3 (29.5–62.8)	10	37.2 (9.2–46.5)	10	21.1	36.2	750.3
8	21/08/2014	58.7 (45.4–63.2)	10	54.3 (6.0–59.8)	10	4.4	7.5	751.1
9	28/08/2014	37.8 (5.2–52.2)	12	24.1 (0.0–37.4)	14	13.7	36.4	761.2
10	11/09/2014	53.8 (34.1–60.0)	18	33.6 (15.2–39.5)	18	20.2	37.5	767.6
11	23/10/2014	33.2 (6.7–45.1)	12	23.2 (2.4–33.1)	12	10.0	30.1	762.8
12	30/10/2014	36.6 (22.7–48.2)	15	32.1 (21.3–40.6)	17	4.5	12.4	766.1
13	28/11/2014	54.0 (33.8–58.7)	12	46.2 (27.3–53.3)	12	7.8	14.3	771.9
14	12/05/2015	43.0 (13.7–58.1)	10	25.7 (0.8–28.7)	12	17.3	40.2	755.4
15	18/06/2015	44.5 (27.2–51.0)	17	34.4 (20.7–41.5)	18	10.1	22.7	751.8
16	19/08/2015	27.7 (8.0–33.8)	15	19.6 (4.2–27.3)	17	8.1	29.4	770.4
17	27/08/2015	47.7 (28.8–56.4)	16	46.9 (29.2–57.4)	17	0.8	1.7	751.1
18	14/10/2015	39.0 (29.1–48.1)	20	40.7 (27.7–48.1)	22	− 1.7	− 4.4	773.4
19	12/11/2015	56.5 (38.9–59.3)	22	39.0 (27.7–50.3)	19	17.5	30.9	753.4
20	19/11/2015	50.3 (33.9–54.6)	17	44.3 (27.7–50.6)	18	6.0	11.8	743.5
21	07/01/2016	38.9 (23.2–43.0)	19	42.0 (32.4–47.5)	18	− 3.1	− 7.9	761.2
	Overall	50.1 (46.0–51.9)	305	41.3 (35.1–44.9)	317	8.8	17.6	

Median (95% CI) percent area loss in the NT and HT groups, and percent neuroprotection in the HT group, across all 21 experiments. The median difference in area loss between the two groups (median NT–median HT) was used to calculate percent neuroprotection in the HT group. Overall median area loss, median difference, and percent neuroprotection were calculated by combining all the animals in the NT (n = 305) and HT (n = 317) groups. The degree of neuroprotection provided by HT was not correlated with initial injury severity (median area loss in the NT group; Kendall’s τb, *p* = 0.83). As a potential factor associated with experimental variability, local barometric pressure on the morning of each experiment is also provided. Data from experiments 7–11 was previously published^6^.

### Animals

All experimental protocols were reviewed and approved by the University of Oslo’s animal ethics research committee. The methods described below were carried out in accordance with those approved protocols, as well as the University of Oslo’s ethical guidelines regarding the use of experimental animals. Within each experiment, 3–7 litters of 8–12 Wistar rat pups of both sexes (33–70 pups per day) underwent unilateral HI. Pups and dams were either bred in house or sourced from external breeders (Charles River, Sulzfeld, Germany; Taconic Biosciences, Ejby, Denmark). Each experiment either used (1) pregnant dams, who were transported to our institution 1 week before their expected due date or (2) litters of sex-balanced pups, which were delivered 24–48 h before the experiment. Before and after HI and treatment, pups were housed with their dams in an animal facility with a 12:12 h dark:light cycle at 21 °C environmental temperature. Food and water were provided ad libitum, and pups were checked for health daily.

### Moderate Vannucci model of unilateral hypoxia–ischaemia

The effects of hypothermia on neonatal HI brain injury were assessed using a modified Vannucci model of unilateral HI in P7 rat pups, as previously described^6,23^. On P7, pups underwent ligation of the left carotid artery under anaesthesia, with 3% isoflurane in a 2:1 gas mixture of NO_2_/O_2,_ via a nose cone. After recovering under a heat lamp, pups were returned to the dams for at least 30 min before being exposed to 8% oxygen for 90–100 min at 36 °C rectal temperature in a specially-designed chamber^6,24^. This length of hypoxia produces a “moderate” injury, with around 30–50% loss of the left hemisphere compared to the right hemisphere in the NT group^6,20^. As both ischaemia and superimposed hypoxia are required to induce brain injury in this neonatal rat model, the right (unligated and contralateral) hemisphere remains undamaged, and can act as an internal control^6,23^. During hypoxia, core temperature was continuously recorded in each chamber in “sentinel” pups carrying a rectal temperature probe (IT-21, Physitemp Instruments, Clifton, NJ, USA). Rectal temperature was maintained within ± 0.2 °C of the target using a servo-controlled water-filled mat (CritiCool, MTRE, Yavne, Israel) inside the chamber, with another animal holding a surface skin temperature probe. Rectal temperatures were manually recorded during all experiments and collated in 10 min epochs. In P7 rats, rectal temperature correlates within 0.1 °C with brain temperature^7^. All surgeries and hypoxia periods occurred between 6 a.m. and 12 p.m. local time on the specified experimental day (Table 1). Local atmospheric pressure during each experimental period was retrospectively collected from www.timeanddate.com and www.yr.no.

### Hypothermia treatment

Before hypoxia, pups were randomised to treatment by litter, weight, and sex. In this model, NT refers to 37 °C, with HT treatment occurring at 32 °C (HT32)^24–26^. Immediately after hypoxia, pups were transferred to chambers at the allotted temperature. Rectal temperature was maintained within ± 0.2 °C of the target, as described above. After 5 h of the allocated treatment, pups were removed from the treatment chambers and returned to the dams^20^.

### Tissue harvesting and processing

Tissue harvesting and processing was performed as previously described^6,20,27–29^. At P14, rats were sacrificed via transcardiac perfusion with saline and 10% neutral-buffered formalin under isoflurane/N_2_O anaesthesia. Brains were harvested and kept in 10% neutral-buffered formalin for 4 days until further processing. Six coronal 3 mm blocks were cut through the brain using a standard rat brain matrix (ASI Instruments Inc., Warren, MI, USA), and embedded in paraffin.

### Area loss analysis

Area loss analysis was performed as previously described^6,20^. For each animal, one 5 μm slide each from the two blocks best representing the cortex, hippocampus, basal ganglia and thalamus were taken (one at the level of the frontal cortex, and one at the mid-hippocampal level), and stained with haematoxylin and eosin (H&E). Slides were scanned (Epson Perfection V750 Pro), and virtual slides were exported as 600dpi images. The optical density and hemispheric area of each section was analysed with ImageJ software (ImageJ, version 1.46r, National Institutes of Health, Bethesda, MD, USA) by an individual who was blinded to group allocation. For each section, the area of each hemisphere was calculated, and the percentage area loss of the left hemisphere relative to the right hemisphere determined by using the following formula: (1 − (area left/area right)) × 100. Two sections were analysed for each animal, and individual area loss was reported as the mean of the area loss across the two analysed sections. In this model, computer-assessed percent hemispheric area loss using this method has previously been shown to be highly correlated with formal neuropathology assessment and specific area and neuronal loss within the hippocampus^6,20^.

### Pathological injury assessment

In order to confirm that area loss analysis accurately reflected formal pathology, a subset of slides underwent pathological scoring by an investigator blinded to treatment allocation. Within each litter from each experiment, the animals displaying the median area loss from the two treatment groups were scored. If there were an even number of animals from a given litter in a treatment group, the two rats around the median were chosen. Using the same slides analysed for area loss, the left side of the brain was examined and four areas of the brain were scored (cortex, basal ganglia, thalamus and hippocampus) by an investigator blinded to the treatment allocation, as previously described^25^ The severity of damage was graded from 0.0 (no injury) to 4.0 (maximum injury, see Thoresen et al.^7^ and Dalen et al.^26^), with intervals of 0.5 for each of the 4 regions, giving a 9-step scale of pathology. Results were analysed based on individual region as well as an average of the scores from these regions, giving a global pathology score.

### Statistical analysis

Only the NT and HT groups from each experiment were used in this analysis. Pups used as rectal and skin temperature probes were also excluded because the stress of restraint has previously been shown to have a neuroprotective effect in this model^30^. Statistical analyses were performed using GraphPad Prism version 8.4 (GraphPad Software, La Jolla, CA, USA), and RStudio version 1.2.5 (RStudio, Boston, MA, USA). Within each experiment, the median difference between groups (with 95% confidence intervals, CI) and percent neuroprotection provided by HT were calculated. The CIs of median differences between groups were calculated according to the method of Bonnet and Price^31^. Percent neuroprotection in the HT group relative to the NT group was calculated with the following formula:

$$Percent\,Neuroprotection=(Median\,NT{-}Median\,HT)/Median\,NT\times 100$$

The presence of severe injury in an individual animal was defined as > 60% area loss, as previously described in an adapted “severe” injury model that included increasing hypoxia time to 150 min, and intra-hypoxic temperature to 37 °C, resulting in a median 60% area loss in the NT group^20^. The median percent area loss, and percent neuroprotection, in the NT and HT groups were calculated by combining the data from all experiments and treating them as single groups of 305 and 317 animals, respectively. Comparison of area loss between the two groups, both overall and split by sex, was performed by calculating the weighted and summed difference of expected versus observed total ranks within each experiment using the Wilcoxon–van Elteren test. Between-group comparisons of regional and global pathology score were carried out with a two-sided Wilcoxon–Mann–Whitney U-test with Bonferroni adjustment for multiple comparisons. Linear regression was used to compare area loss with global pathology score. For overall area loss and global pathology score a Cohen’s d effect size (ES) was calculated using the Mann–Whitney U statistic and transformation of η^2^ according to the methods of Lenhard and Lenhard^32–34^. To determine how many animals might be required in each group to find the overall ES of HT detected here, a post hoc power calculation was performed using G*Power version 3.1^35^.The ES was entered into G*Power, along with the following parameters: α = 0.05, β = 0.8, comparison test = two-tailed Wilcoxon–Mann–Whitney U-test. This resulted in a predicted number of animals that would be required in each group to detect the corresponding ES. Within the NT and HT groups, Spearman’s r correlation matrices were constructed to examine the association between experimental factors and area loss. Experimental factors that were significantly associated with area loss in these unadjusted correlations were then entered into two logistic regression models to predict dichotomous outcome: (1) whether an animal in the NT group had area loss greater than the median area loss across all experiments, and (2) whether an animal in the HT group experienced neuroprotection (e.g. had an area loss below the median area loss in the NT group within that experiment). Regression models are reported with parameter estimates and 95% CI, as well as overall accuracy. Kendall’s tau-b (τb) correlation coefficient was calculated when examining the effect of individual experimental variables on percent area loss and neuroprotection by HT. A two-sided *p* value < 0.05 was considered statistically significant.

## Results

### Included data

Data from a total of 622 animals from 106 litters (NT, n = 305; HT, n = 317 animals) were included in the final analysis. Median (range) NT or HT group size in each individual experiment was 14.5 (10–22) animals. In addition to area loss assessment, a total of n = 277 (44.5%) of animals underwent formal pathological scoring. These were selected as the 1–2 animals representing the median NT and HT area loss in each litter within each experiment. The median number of litters in each experiment was n = 5 (range n = 3–7). Animal characteristics from each experiment (numbered in chronological order) are summarised in Supplemental Table S1.

### Area loss and hypothermic neuroprotection after moderate HI

**Figure 1:**
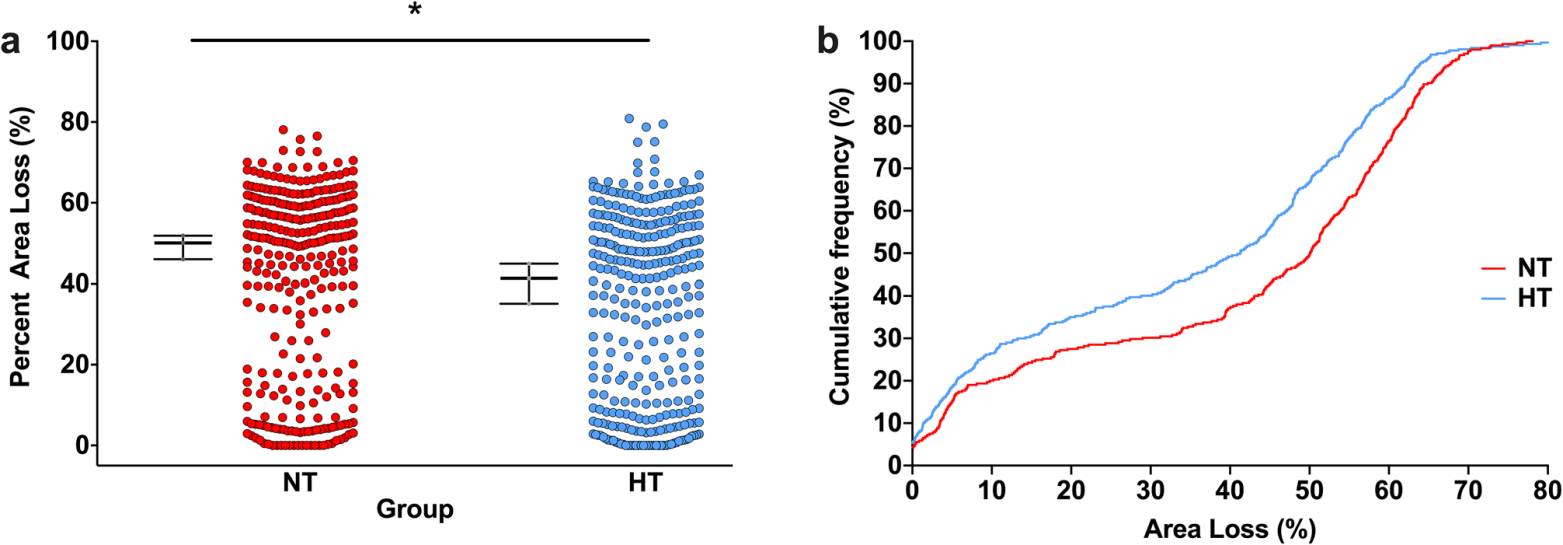
Hypothermic neuroprotection. (**a**) Scatter plot of percent area loss in the NT (n = 305) and HT (n = 317) groups. The median with 95% CI is plotted adjacent to each scatter. Across the 21 experiments, median (95% CI) area loss in was 50.1% (46.0–51.9%; n = 305) in the NT group, and 41.3% (35.1–44.9%; n = 317) in the HT group. *Denotes significant neuroprotection (*p* < 0.001). (**b**) Cumulative frequency plot from the NT (n = 305) and HT (n = 317) groups, with each observation resulting in a proportional increase in the y-axis. A relatively uniform degree of neuroprotection is seen across the whole range of injury severity, particularly between 10 and 60% area loss.

**Figure 2:**
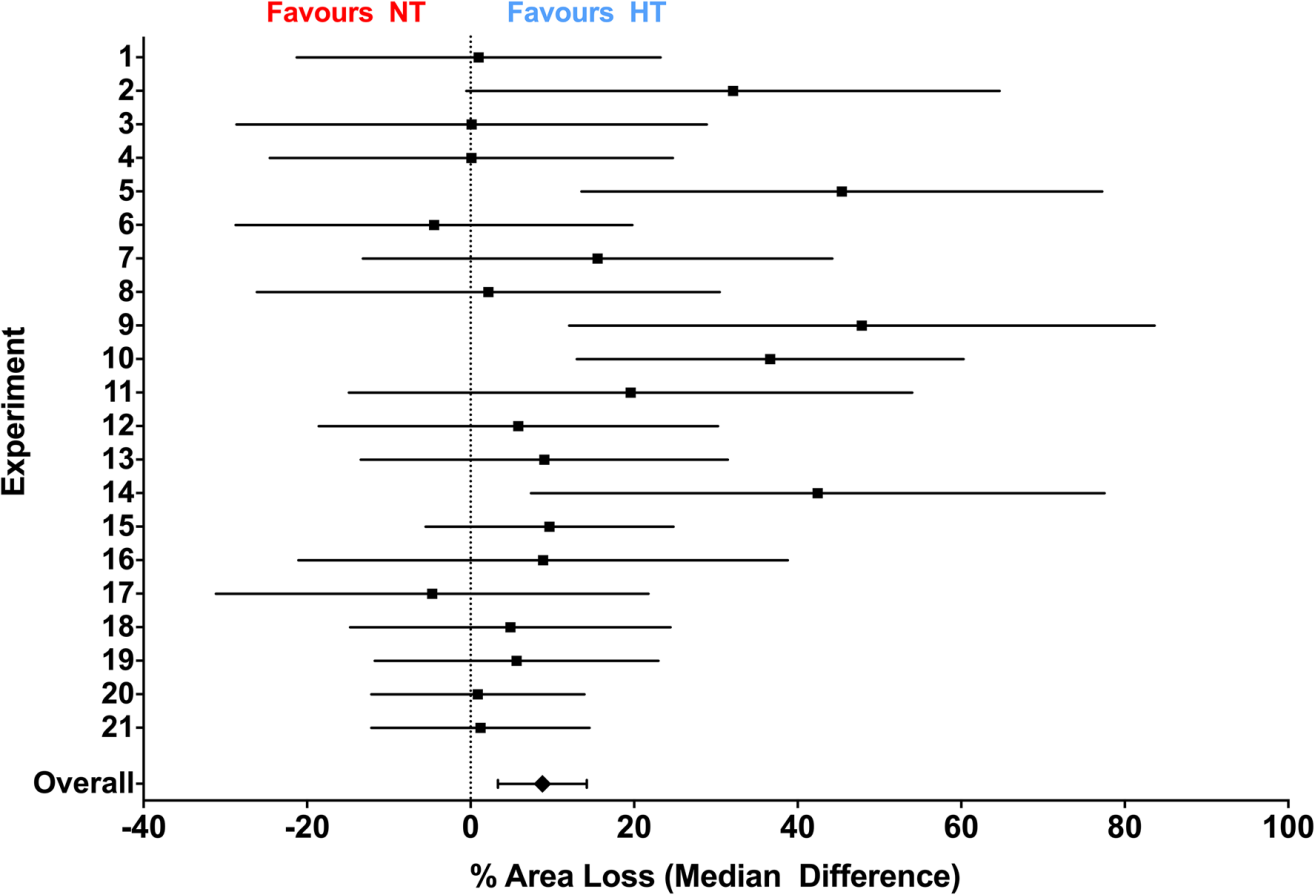
Median difference in area loss between NT and HT. Median difference (with 95% CI) in area loss between the NT and HT groups in each individual experiment, and overall. Experiments are numbered in chronological order. Of the 21 experiments, 15 favoured HT, and the overall median difference (absolute reduction in area loss in the HT group) was 8.8% (3.4–14.2%).

Across the 21 experiments, median (95% CI) area loss was 50.1% (46.0–51.9%; n = 305) in the NT group, and 41.3% (35.1–44.9%; n = 317) in the HT group (Fig. 1a). Plotting the cumulative frequencies in each group with increasing area loss suggested a relatively uniform degree of neuroprotection across the whole range of injury severity, particularly between 10 and 60% area loss (Fig. 1b). For each experiment, the median area loss in the NT and HT groups, median difference between the two groups, and percent neuroprotection in the HT group, is listed in Table 1. Median difference (NT–HT) area loss in each individual experiment ranged from − 13.9 to 21.1%, with an overall median difference (95% CI) of 8.8% (3.4–14.2%) between the two groups (Fig. 2). Percent neuroprotection (median difference as a percentage of area loss in the NT group) ranged from − 92.4 to 42.7%, with an overall median of 17.6% (6.8–28.3%) neuroprotection in the HT group compared to the NT group. Within each experiment, the degree of neuroprotection provided by HT was not correlated with initial injury severity (median area loss in the NT group; Kendall’s τb = 0.038, *p* = 0.83). HT appeared to be similarly neuroprotective across the range of injury, with the NNT (number needed to treat) to prevent an incidence of > 60%, > 50%, > 40%, > 30%, and > 20% areas loss being 10.0, 5.8, 8.2, 9.8, and 12.8, respectively.

### Regional and global pathology scoring

**Figure 3:**
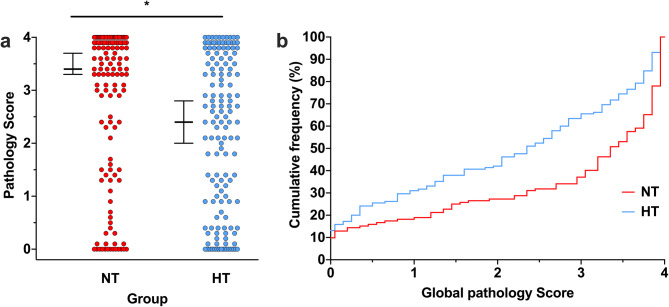
Global pathology scoring. (**a**) In the subset of animals that underwent formal pathology scoring (n = 277; n = 132 NT, n = 145 HT), median (95% CI) global pathology score 3.4 (3.3–3.7) in the NT group, and 2.4 (2.0–2.8) in the HT group. *Denotes significant neuroprotection (*p* < 0.001). (**b**) Cumulative frequency plot from the NT (n = 132) and HT (n = 145) groups, with each observation resulting in a proportional increase in the y-axis. A relatively uniform degree of neuroprotection is seen across the whole range of injury severity, with increasing relative neuroprotection in the HT group up to a global pathology score of 3.0.

In the 44.5% of animals that underwent formal pathology scoring (n = 277; n = 132 NT, n = 145 HT), median (95% CI) global pathology score was 3.4 (3.3–3.7) in the NT group, and 2.4 (2.0–2.8) in the HT group (*p* < 0.001; Fig. 3a). Similar to area loss, plotting the cumulative frequencies in each group with increasing pathology showed neuroprotection across the whole range of injury severity, with the greatest difference between groups at a global pathology score of 3.0 (Fig. 3b). Regional pathology scores in the NT group were 4.0 (3.5–4.0) in the hippocampus, 3.5 (3.0–4.0) in the thalamus, 3.0 (2.5–3.5) in the basal ganglia, and 3.65 (3.3–3.8) in the cortex (Supplemental Figure S1). Significant neuroprotection was seen in all regions in the HT group, with corresponding scores of 2.0 (2.0–3.0) in the hippocampus (*p* < 0.001), 2.5 (2.0–3.0) in the thalamus (*p* = 0.007), 2.0 (1.5–2.0) in the basal ganglia (*p* = 0.001), and 3.0 (2.3–3.0) in the cortex (*p* = 0.003).

### Area loss versus global pathology scoring—effect size and power analysis

**Figure 4:**
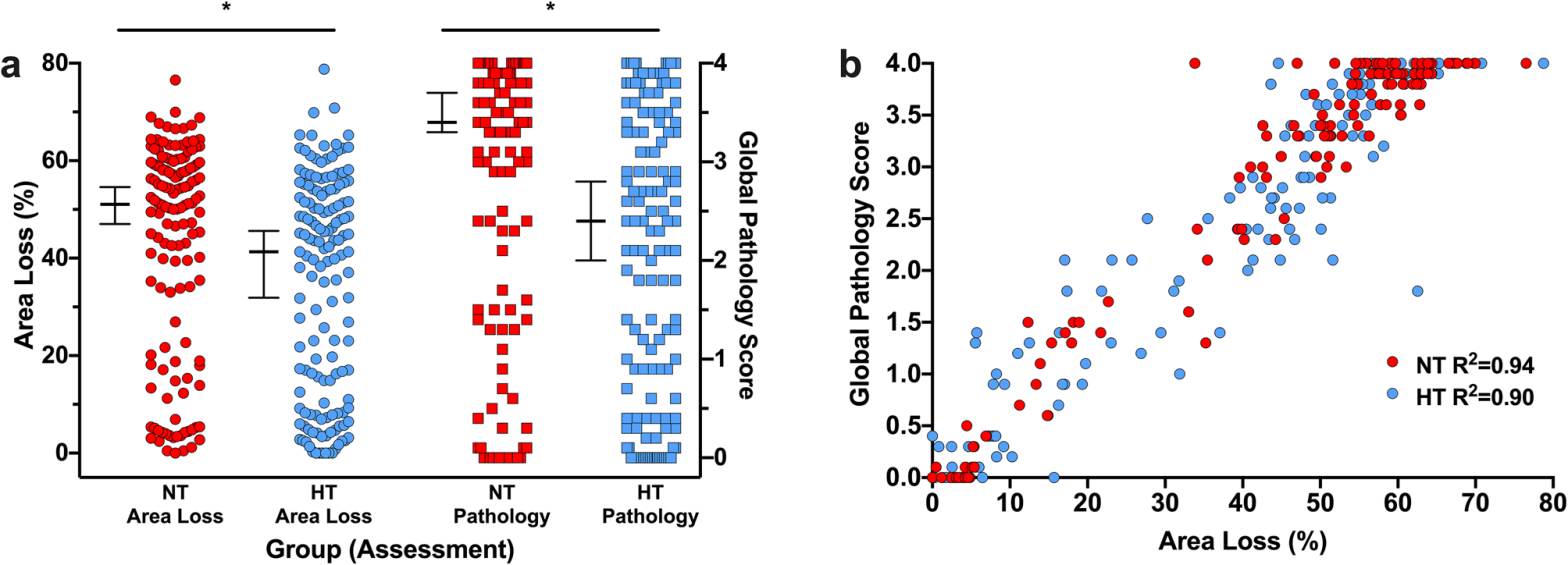
Comparison between area loss and global pathology score. (**a**) Direct comparison of area loss and pathology score in the n = 277 (n = 132 NT, n = 145 HT) animals that underwent pathology scoring, representing the median injury in the NT and HT groups within each litter from each experiment. Median (95% CI) area loss was 51.0% (47.0–54.6%) in the NT group, and 41.3% (31.6–45.6%) in the HT group, with corresponding global pathology scores of 3.4 (3.3–3.7) in the NT group, and 2.4 (2.0–2.8) in the HT group. This resulted in a 19.0% neuroprotection by HT, compared to 25% neuroprotection when comparing the same animals using global pathology score. The resulting ES for HT was 0.42 when using area loss, and 0.51 when using global pathology. (**b**) Global pathology score was linearly correlated with area loss in both the NT (R^2^ = 0.94, *p* < 0.001) and HT (R^2^ = 0.90) groups (*p* < 0.001 for both).

The overall Cohen’s d ES for the effect of HT on area loss was 0.29 in the entire cohort. Assuming a non-normal parent distribution, α = 0.05, β = 0.2 and balanced group sizes, n = 218 animals per group would be required to detect the true ES of HT on area loss. In the subset of animals that underwent pathology scoring, median (95% CI) area loss was 51.0% (47.0–54.6%) in the NT group, and 41.3% (31.6–45.6%) in the HT group (Fig. 4a). In animals analysed for both area loss and pathology, HT therefore resulted in 19.0% neuroprotection when analysed using area loss, which was increased to 25% neuroprotection when comparing the same animals using global pathology score. The corresponding ES for HT was 0.42 when using area loss, and 0.51 when using global pathology, resulting in a predicted n = 105 and n = 71 animals per group being required to detect the ES using area loss and pathology, respectively. Global pathology score was linearly correlated with area loss in both the NT (R^2^ = 0.94, *p* < 0.001) and HT (R^2^ = 0.90) groups (Fig. 4b).

### Experimental variables

The variability in injury of the Vannucci model is well-documented^6,9^. To explore factors that might be associated with experimental variability, Spearman’s rank correlation matrices were constructed for both the NT and HT group to assess whether individual area loss was significantly associated with any of the following variables: sex, weight at P7, ligation time, delay between ligation and hypoxia, litter size (number of animals in the litter after HI until P14), and barometric pressure during HI (Supplemental Figures S2A and S2B). Median (range) barometric pressure during HI was 761.2 mmHg (743.5–773.4 mmHg). In both the NT and HT groups, barometric pressure during HI displayed a small but significant association with area loss (Supplemental Fig. 3A). The magnitude of the association was similar in both groups (Kendall’s τb: NT = −0.105, *p* = 0.008; HT = −0.109, *p* = 0.005), with higher barometric pressures associated with lower area loss. When grouped by quintiles of intra-experimental barometric pressure (n = 58–68 animals per group per quintile), a nadir of area loss was seen in both groups in the 4th quartile (763.5–769.6 mmHg; Supplemental Fig. 3B). However, barometric pressure during HI was not associated with degree of individual neuroprotection provided by HT as measured by each HT animal’s percent neuroprotection relative to the median NT area loss for that experiment (Kendall’s τb = 0.026, *p* = 0.5). In the NT group only, litter size was inversely associated with area loss (Kendall’s τb = −0.151, *p* < 0.001; Supplemental Fig. 3C), with P7 weight associated with area loss in the HT group only (Kendall’s τb = 0.081, *p* = 0.03; Supplemental Fig. 3D).

Median (IQR) carotid ligation time (period under anaesthesia during ligation) was 6 (5–6) minutes in the NT group, and 6 (5–6) minutes in the HT group. Ligation time did not affect area loss (Data not shown). Median (IQR) time between ligation and hypoxia was 119 (95–146) minutes in the NT group, and 122 (95–146) minutes in the HT group. Delay between ligation and hypoxia was not correlated with injury or effect of hypothermia (Supplemental Figure S3E). No correlation between the source (vendor) of the animals and the individual experimental outcomes were seen. Weight at P7 and P14 was similar in both groups, as was total weight gain (data not shown). An inverse correlation between weight gain and area loss at P14 was seen in both the NT and HT groups (Kendall’s τb: NT = −0.257, HT = −0.316; *p* < 0.01 for both; Supplemental Figure S3F). Rectal temperature monitoring indicated that in all experiments target temperatures were achieved within 10 min and remained stable throughout (Supplemental Figure S4).

Barometric pressure and parameters associated with area loss in the correlation matrixes—either litter size (NT group) or P7 weight (HT group)—were entered into two logistic regression models to predict: (1) whether an animal in the NT group had area loss greater than the median area loss across all experiments (50.1%), and (2) whether an animal in the HT group experienced neuroprotection (had an area loss below the median area loss in the NT group within that experiment). The regression for the NT group was able to predict above median area loss with an accuracy of 60.3% (Supplemental Table S2). No model was able to predict neuroprotection in HT animals. Based on our aggregated data, variability of the neuroprotective effect of HT could therefore not be explained by basic experimental variables.

### Effect of sex on area loss after HT treatment

**Figure 5:**
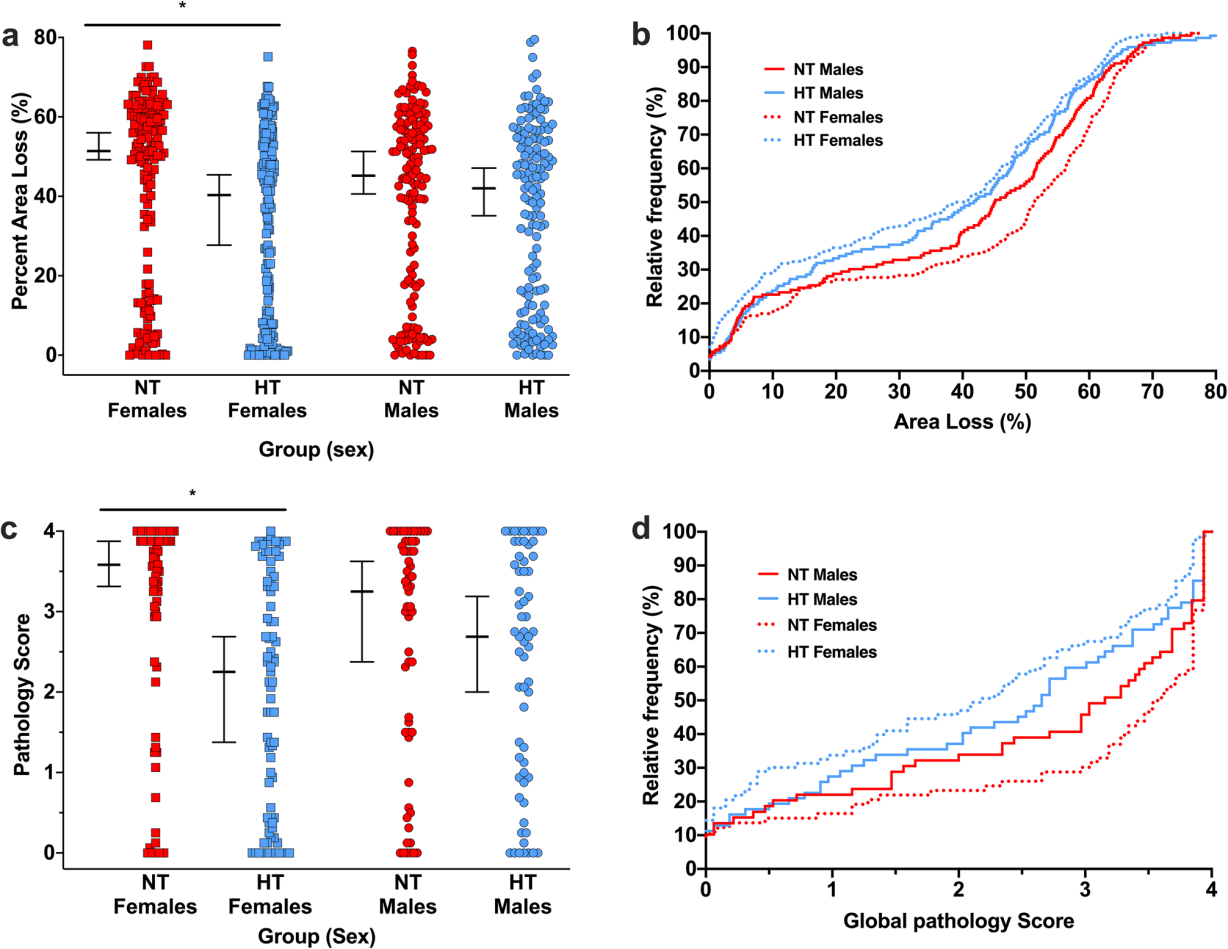
Sex effects of hypothermic neuroprotection. (**a**) Median (95% CI) area loss in the NT group was 51.4% (49.2–56.0%; n = 159) in females, and males 45.2% (40.6–51.3%; n = 146) in males. In the HT group, median area loss was 40.3% (27.7–45.4%; n = 170) in females, and 42.0% (35.1–47.1%; n = 147) in males. The median differences between the NT and HT groups were 11.1% (21.6% neuroprotection) in females, and 3.2% (7.1% neuroprotection) in males. (**b**) Cumulative frequency distribution plot of area loss. (**c**) Median (95% CI) global pathology score in females was 3.6 (3.3–3.9, n = 73) in the NT group, and 2.3 (1.4–2.7, n = 83) in the HT group. In males, median global pathology score was 3.3 (2.4–3.6, n = 59) in the NT group, and 2.7 (2.0–3.2, n = 62) in the HT group. (**d**) Cumulative frequency global pathology score. The cumulative frequency plots suggest HT is neuroprotective across the entire range of injury in females, with neuroprotection in males centred solely around moderate injury scores (~ 20–55% area loss). *Denotes significant neuroprotection (*p* < 0.001).

Within each individual experiment, no effect of sex was seen with regard to degree of damage or neuroprotective effect of HT. However, the picture was distinctly different once all the data was analysed together. Median (95% CI) area loss in the NT group was 51.4% (49.2–56.0%; n = 159) in females, with a trend (*p* = 0.07) towards lower injury in males (median 45.2%, CI 40.6–51.3%; n = 146). In the HT group, median area loss was 40.3% (27.7–45.4%; n = 170) in females, and 42.0% (35.1–47.1%; n = 147) in males. The median differences between the NT and HT groups were therefore 11.1% (21.6% neuroprotection) in females, and 3.2% (7.1% neuroprotection) in males. The overall significant neuroprotective effect of HT appeared to be due to a highly significant neuroprotective effect in females (*p* < 0.001), which compensated for a non-significant effect in males (*p* = 0.07; Fig. 5a). A similar pattern was seen when comparing the animals who underwent pathology scoring. In female animals, median (95% CI) global pathology score was 3.6 (3.3–3.9, n = 73) in the NT group, and 2.3 (1.4–2.7, n = 83) in the HT group (*p* < 0.001, Fig. 5c). In males, median global pathology score was 3.3 (2.4–3.6, n = 59) in the NT group, and 2.7 (2.0–3.2, n = 62) in the HT group, which was not significantly different (*p* = 0.2, Fig. 5c). Cumulative frequency distribution plots of both area loss (Fig. 5b) and global pathology score (Fig. 5d) also show that HT is neuroprotective across the entire range of injury in females, with neuroprotection in males centred around moderate scores (~ 20–55% area loss). In female animals, HT was significantly neuroprotective in all regions (hippocampus, thalamus, basal ganglia, cortex), but no region-specific neuroprotection was seen in males (data not shown).

### Distribution and consistency of injury

The version of the Vannucci model described here is expected to result in a median area loss of around 40% in the NT group^6^. Only 3/21 experiments displayed median area loss in the NT group below 30% (experiments 2, 4, and 16), and two of those had over 25% median area loss in the NT group, suggesting relatively consistent injury across experiments. On visual inspection of the combined data from all 622 animals (Fig. 1), a distinct bimodal distribution of injury was seen, with 87.9% of all animals (547/622) displaying a percent area loss that was either > 40% or < 20%. In the NT group, 192 of 305 (62.5%) animals had > 40% area loss, and 83 (27.0%) had < 20% area loss. In the HT group, 161 of 315 (51.1%) animals had > 40% area loss, with 111 (35.2%) having < 20% area loss. HT resulted in a 16.7% absolute decrease in percentage of females with > 40% injury, from 66.7% (NT females) to 50% (HT females), compared to a 7.2% reduction in males (58.9% of NT males to 52.7% of HT males). The percentage of animals with < 20% injury also increased by a greater extent in females (10.1%, from 26.4% to 36.5%) compared to males (5.2%, from 28.1% to 33.3%). The same bimodal injury pattern was seen in the animals analysed for global pathology, with the majority of animals having either high (2–4) or low (0–0.5) pathology scores within in each brain region (Supplemental Figure S1).

## Discussion

This study provides a novel approach to analysis and reporting of data from a single laboratory and animal model. Summarising the data from all our experiments employing HT treatment in the standard Vannucci model of moderate HI injury enables us to overcome what Robert Rosenthal termed the “file drawer problem”^36^, and get greater insight into the model itself. He remarked that “for any given research area, one cannot tell how many studies have been conducted but never reported”. This is not something that can be solved with a priori power calculations and meta-analyses of published studies^22^. As with this study, the file drawer problem is likely to be particularly pertinent to unpublished pilot studies. The majority of the work summarised here is from pilot studies that also included non-HT treatment groups that would otherwise have remained unpublished.

Producing animal models that accurately replicate human disease and provide robust platforms for translating treatments to the clinic is a notoriously difficult challenge for modern medicine^2,37^. One area where this is particularly relevant, and has been extensively discussed, is in the translation of neuroscience to the treatment of neurological disease^2,22,38,39^. Due to the typically-small group sizes and low statistical power seen in most preclinical neuroscience studies, even positive findings are likely to be greatly exaggerated^22^. This may be a major reason why, when examining experimental treatments for acute stroke, O'Collins et al. found little correlation between treatment effect in animal models and subsequent translation to clinical trials in humans^38^.

In the field of neuroprotection, the benefit of HT for asphyxiated term infants with moderate-to-severe encephalopathy is one of the few successes to date^3^. However, the degree of neuroprotection does not appear to directly translate from published animal work. In preclinical studies of HT after neonatal HI, successful HT typically provides around 40–65% neuroprotection^6,7,9,12,40,41^. Though the formal pathology scoring used preclinically is not directly comparable to outcomes (mortality and neurodevelopmental disability) in children, the magnitude of the previously described preclinical effect of HT appears to be significantly greater than that seen in clinical trials^3^. A large part of this discrepancy may be due to the methodological issues associated with cooling asphyxiated neonates within the optimal time window for neuroprotection where, compared to standardised animals models, the exact timing, mechanism, and duration of injury in infants with HIE is often unknown^42,43^. This includes the heterogeneous clinical scenarios that may contribute to HIE risk including infection, pre-eclampsia, gestational diabetes, placental pathologies, intrauterine growth restriction and even maternal stress, in addition to a perinatal sentinel event^44–50^. We found that HT was significantly neuroprotective when administered immediately after HI, though the effect was variable across experiments and the overall neuroprotective effect was smaller than reported in some previous studies^51^. This is to be expected once the overall size of the treatment groups increases^22^. Importantly, the injury in the NT group was consistent (> 25% area loss in 20/21 experiments), suggesting that the majority of the variability was due to different responses to HT itself within each experiment.

One strength of our approach is that the size of the dataset allows for the exploration of a number of factors that may influence the degree of injury seen and the response to HT. Interestingly, barometric pressure during the morning (surgical and HI period) of each experiment was associated with the degree of injury seen in both the HT and NT groups. Though higher barometric pressure tended to result in lower injury, the nadir of injury occurred in the 4th quintile of pressure for both groups. However, barometric pressure was not able to accurately predict whether animals would experience greater than average injury in the NT group, and was not significantly associated with the degree of neuroprotection seen in the HT group. Though P7 weight in the HT group was negatively associated with area loss, no clear experimental, operator, or environmental factor could be identified that corresponded with experiments where HT was not neuroprotective or the overall degree of neuroprotection afforded by HT. We and other have previously shown that individual anatomical factors such as cerebral blood flow during HI can significantly affect the final degree of injury^52,53^. However, while these individual differences are likely to contribute to the variability and pattern of injury seen in the model, they are likely to average out with randomisation procedures such as those used in our studies. This suggests that as-yet unidentified factors will alter the effect of HT (and potentially other therapies) in a given preclinical experiment, and this variability should be expected during experimental planning.

Overall, HT resulted in 17.6% neuroprotection of area loss in the HT group, with a corresponding treatment ES of 0.29. In the 277 animals who underwent pathology scoring (the 1–2 animals with median area loss in the NT and HT groups within each litter), the ES of HT was 0.42 when comparing area loss, and 0.51 when using formal pathology scoring, suggesting a small-to-moderate effect of HT on severity of injury^34^. Using formal pathology scoring would reduce the number of animals required to see the true ES by at least 30%. Though pathology score is more sensitive to the effects of HT at the cellular level, it is interesting to note that the animals with the highest pathology scores (3.5–4.0) had a wide range of area loss (40–70%), and a combination of the two methods may be ideal when assessing the effects of a therapy in more severe injury. Importantly, both cumulative frequency plots and dichotomous cut-offs at 20%, 30%, 40%, 50%, and 60% area loss suggested that HT was similarly neuroprotective regardless of severity of injury, though the effect does appear to be lost at the upper limit of severity (> 70% area loss or global pathology score > 3.5). While preclinical studies are not directly comparable to clinical trials, the overall neuroprotective results in the our model are very similar to those seen for the ~ 15% absolute reduction in death and major disability (RR 0.75, 0.68–0.83; NNT = 7) in the largest meta-analyses of clinical trials to date^3,54^.

The reduction in more severe injury by HT in this analysis initially appears at odds to our previous work in this model, where we have found multiple times that HT is not neuroprotective after severe injury resulting in a median area loss of ~ 60%^6,20^. However, the modification of the insult that resulted in this severe injury included increasing the insult temperature from 36 to 37 °C, and increasing the hypoxia time to 150 min^6,20^. Another modification to the insult, using the same hypoxia parameters described in this meta-analysis but pre-exposing pups to the Toll-like receptor 2 agonist PAM_3_CSK_4_, also resulted in 60% median area loss in the NT group, but significant neuroprotection in the HT group was seen^29^. By comparison, pre-sensitisation with *E. coli* lipopolysaccharide negates the neuroprotective effect of HT in this model, despite only “moderate” levels of injury^28,29^. This suggests that the mechanism of the insult itself (including inflammatory stimulus, temperature, and hypoxia time) is likely to dictate whether HT is neuroprotective or not, and not the absolute degree of injury.

As is becoming increasingly reported in the preclinical and clinical literature, a sex difference in response to HT was also seen, with a much greater therapeutic effect in females. Previously, when we compared area loss or neuropathology scores as outcomes after HI, these have largely been equivalent between the sexes^6,20,24,55^, though we have seen improved functional outcomes in female animals based on neurobehavioural testing^25,55^. This sexually-dimorphic effect of HT in our model, and greater benefit in females, is also in line with recent work from other groups^56–58^. This result is likely to be due to the effects of HT on the variety of cell death pathways initiated after cerebral HI. Females appear to more specifically rely on the classical apoptotic pathways of cells death, with HT thought to inhibit the apoptotic cascade at multiple levels^59–61^. Conversely, male cells appear to die via non-apoptotic pathways such as *parthanatos* caused by the depletion of cellular NAD + due to the activation of PARP-1 (Poly [ADP-ribose] polymerase 1)^60,62^*.* As a result of sexually-dimorphic mechanisms of injury and inflammatory responses, a number of therapies have been found to only benefit one sex or the other, and this is an important area for significant future research in the neonatal neuroprotection field^63^.

Considering our results and the historical skew towards the use of male animals in neuroscience to minimise variability as a result of the oestrus cycle, it is possible that similar results in other models of brain injury may well have prevented some neuroprotective strategies from being fully investigated^64^. The large group sizes used here might have promoted a regression towards the mean that would also be seen if other studies showing improved outcomes in males treated with HT were repeated with more animals. However, it is also important to note that rodent studies of HT usually only include a formal HT period of up to 5 h, with some natural ongoing hypothermia (rectal temperature < 36 °C) also likely to occur in the nest afterwards^6,27^. As temperature control is generally poorly-reported in rodent neuroprotection studies^65^, comparing the dose an timing of HT to that seen in humans is difficult. Despite this, the largest clinical trials of HIE do not appear to have definitively questioned whether any outcome parameters in asphyxiated infants treated with HT are affected by sex^3,17,18,56,66^. This information is crucial to the ongoing effort to refine neuroprotective strategies for infants with HIE, and with further investigation it may be found that male infants respond less well to standard HT treatment. Due the differing mechanisms of injury seen in response to an insult in males and females^60^, it is likely that different treatment strategies will prove beneficial based on sex as well as underlying mechanism of injury. Males may benefit from a different HT temperature or cooling strategy compared to females^67^, or see greater benefit after the addition of adjunct therapies. For instance, Park et al. found that HT was neuroprotective in combination with mesenchymal stem cells (MSCs) in a pre-selected group (> 50% hemispheric involvement on MRI) of male rats after HI at P7^68^. By contrast, Herz et al. found that adding MSCs to HT after unilateral HI in a sex-balanced unselected group of P9 mice was deleterious^69^. Male animals may also benefit from receiving a different treatment strategy altogether, potentially including sex hormone therapies such as progesterone^70^, or prolonged or multi-pronged treatments that target oxidative stress pathways^58,71^, or the replenishment of NAD + (oxidised nicotinamide adenine dinucleotide)^62^.

Another important aspect of the model that becomes increasingly clear as data is aggregated from multiple experiments is the distribution of injury. Two peaks of injury are seen, one above 40% area loss and the other below 20% area loss. This suggests two discrete populations of animals with underlying differences that determine the initial degree of injury, for instance due to variability in anatomical and physiological blood flow during the insult^52,53^. Treatment with HT resulted in a 16.7% decrease in percentage of females with > 40% injury, which was greater than the 7.2% reduction in males. The percentage of animals with < 20% injury also increased by a greater extent in females (10.1%) compared to males (5.2%). This suggests that HT is able to reverse the majority of injury in a certain subset of animals initially destined to have > 40% injury, but that this effect is more likely to occur in females. More importantly, however, is the way in which the underlying distribution of injury should guide analysis of data from this model. It should be assumed that all data from the model must be analysed using non-parametric methods, with the bimodal distribution of injury suggesting that most studies in this model are dramatically underpowered to detect true treatment effects. Indeed, the analysis presented here is only possible due to the much larger group sizes generally presented in preclinical studies. For instance, the protocol for the “Optimizing Cooling for Neonatal (HIE)” trial cited a number of preclinical studies, with typical treatment group sizes of 4–17 animals^66,72^, and the largest single treatment group of all of the referenced preclinical studies including 31 rats^25^. These experimental numbers are in stark contrast to the 726 infants that were planned to be included in the trial before it was stopped at the halfway point due to lack of likely benefit in the experimental groups, and a potential for increased harm^66^. While the initial clinical trials of HT were largely based on that same body of animal work, the optimisation of an already beneficial treatment protocol will involve the elucidation of much smaller relative effect sizes, which necessitates larger experimental groups^22^. Therefore, though the three R’s (Replacement, Refinement, and Reduction) are essential to improving the quality and sustainability of preclinical research^2^, the improved use of animal resources must include ensuring that group sizes are large enough to produce adequately-powered results that are more likely to translate to the clinic. Indeed, our analysis suggests that to see the true ES of HT in this model, more than 100 animals per group may be required depending on the outcome measured.

Our study does have several limitations. Compared to the global injury and multi-factorial aetiology of neonatal HIE, the unilateral brain-focused injury investigated here cannot fully replicate the complex pathologies and systemic organ involvement seen clinically. As mentioned above, we also only performed short-term survival with formal pathological scoring in a representative subset of animals. However, this allowed us to examine the effects of HT in multiple experiments and in a large number of animals, using an outcome measure (area loss) that correlates highly with formal neuropathology. This information then enables us to refine the parameters of the model to look for neuroprotective treatments for specific subgroups (i.e. males). As the field moves forward, the variability of the Vannucci model clearly suggests that much larger group sizes than have been traditionally used should be employed to investigate potential treatments. For in-depth analyses, this will likely require collaboration between multiple expert research groups, as well as the promotion of avenues for the collation and publication of pilot data^22^. To increase the likelihood of successful translation, any promising therapy would then be repeated using larger groups sizes and long-term pathological and behavioural assessments^27,55^, ideally in multiple laboratories. This would then inform work in larger animals such at the piglet or fetal sheep^73^. As the pipeline applied to promising therapies for term neonatal HIE is fairly robust, one of the most important areas for development is the implementation of significantly robust studies in rodents such that later work in larger models is focused on the most promising therapies.

While one benefit of the large aggregated dataset explored here is the ability to draw on increased statistical power and eliminate some of the effects of inter-experiment variability, it must be acknowledged that each individual experiment employed only 10–22 animals per treatment group. As such, it is potentially problematic to then suggest that several dozen animals are required per group in future studies examining treatment effects in the Vannucci model. However, the main purpose of a meta-analysis is to gain greater insight into a particular disease or treatment than is possible from each individual smaller study or trial. With respect to future work, it is worth nothing that finding the true effect size of a therapy in a model is not necessarily the same as screening for whether or not a therapy has some degree of neuroprotective effect. For the latter purpose, using smaller group sizes (n = 20–30 per group) with animals combined from experiments performed over a short period is likely to remain worthwhile. The previously published experiments included in this analysis (experiments 7–11) are an example of this, where multiple treatment temperatures were assessed in the model^6^. These experiments were performed over a 2-month period, with each experiment including four of the six final experimental groups. In these experiments, injury was fairly variable (37.5–60.7% median area loss in NT group), but the average degree of neuroprotection by HT was relatively stable. In four out of five experiments neuroprotection by HT was 30.1–37.5%, with 7.5% neuroprotection in the fifth. Importantly, every experiment included a standard NT injury group and standard HT treatment group as they are defined in this manuscript. This enabled us to more confidently combine experiments as each had control groups for both injury (NT) and treatment (HT). Including control groups for both injury and treatment in all experiments, and randomising across all treatment groups in all experiments, should be the standard approach. This is particularly important if multiple experiments have to be combined to adequately power an overall study. These studies should also be powered to detect the presence of sex-based differences (therefore at least doubling the target group size), and non-parametric statistical analysis methods should be used exclusively unless a significant body of outcome data can be shown to be normally distributed.

In conclusion, we present our most recent body of work, including all pilot data, examining the neuroprotective effect of HT in the Vannucci model of unilateral HI brain injury in P7 rats. Hypothermia provided a smaller overall neuroprotective effect than previously reported in this model, but this result more accurately reflects those seen from clinical trials. The main neuroprotective effect of HT derived from a greater effect in females, and future work must be performed to investigate the mechanisms behind the failure of HT in certain scenarios. As this model has translated successfully to other animals and clinical HT treatment, it provides a useful potential starting point to investigate treatments for subgroups of asphyxiated infants based on sex and severity of injury.

## Supplementary information

Supplementary information
