# Supplementary material for: Variability and sex-dependence of hypothermic neuroprotection in a rat model of neonatal hypoxic–ischaemic brain injury: a single laboratory meta-analysis
Source: Sci Rep. 2020 Jul 2;10:10833. doi: 10.1038/s41598-020-67532-2 (PMC7331720; doi:10.1038/s41598-020-67532-2)
Supplement: Supplementary file 1 — Supplementary information [file 41598_2020_67532_MOESM1_ESM.docx]

**Variability and sex-dependence of hypothermic neuroprotection in a rat model of neonatal hypoxic-ischaemic brain injury: a single laboratory meta-analysis**

Thomas R. Wood, Julia K. Gundersen, Mari Falck, Elke Maes, Damjan Osredkar, Else Marit Løberg, Hemmen Sabir, Lars Walløe, Marianne Thoresen*.

**Correspondence:** marianne.thoresen@medisin.uio.no

**SUPPLEMENTARY DATA**

**
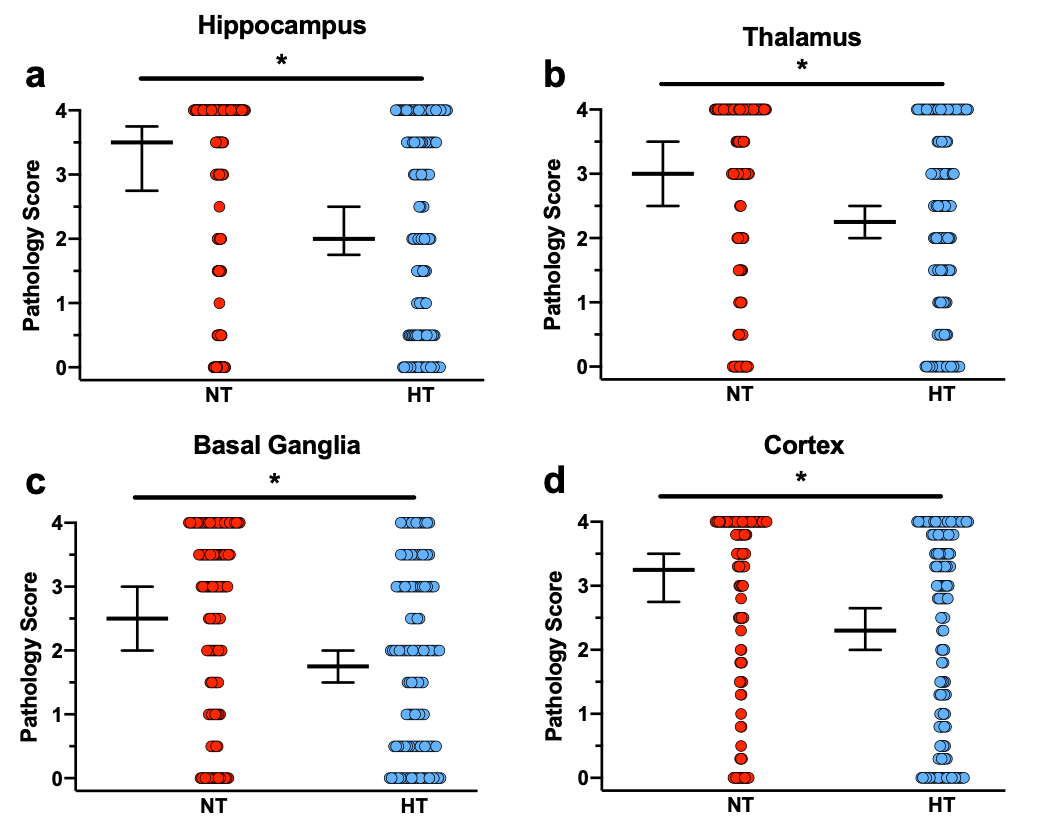
**

**Supplemental Figure S1. Regional pathology scoring.** In the subset of animals that underwent formal pathology scoring (n=277; n=132 NT, n=145 HT), median (95% CI) regional pathology scores in the NT group were (**A**) 4.0 (3.5-4.0) in the hippocampus, (**B**) 3.5 (3.0-4.0) in the thalamus, (**C**) 3.0 (2.5-3.5) in the basal ganglia, and (**D**) 3.65 (3.3-3.8) in the cortex. Significant neuroprotection was seen in all regions in the HT group, with corresponding scores of 2.0 (2.0-3.0) in the hippocampus, 2.5 (2.0-3.0) in the thalamus, 2.0 (1.5-2.0) in the basal ganglia, and 3.0 (2.3-3.0) in the cortex. * denotes p<0.05


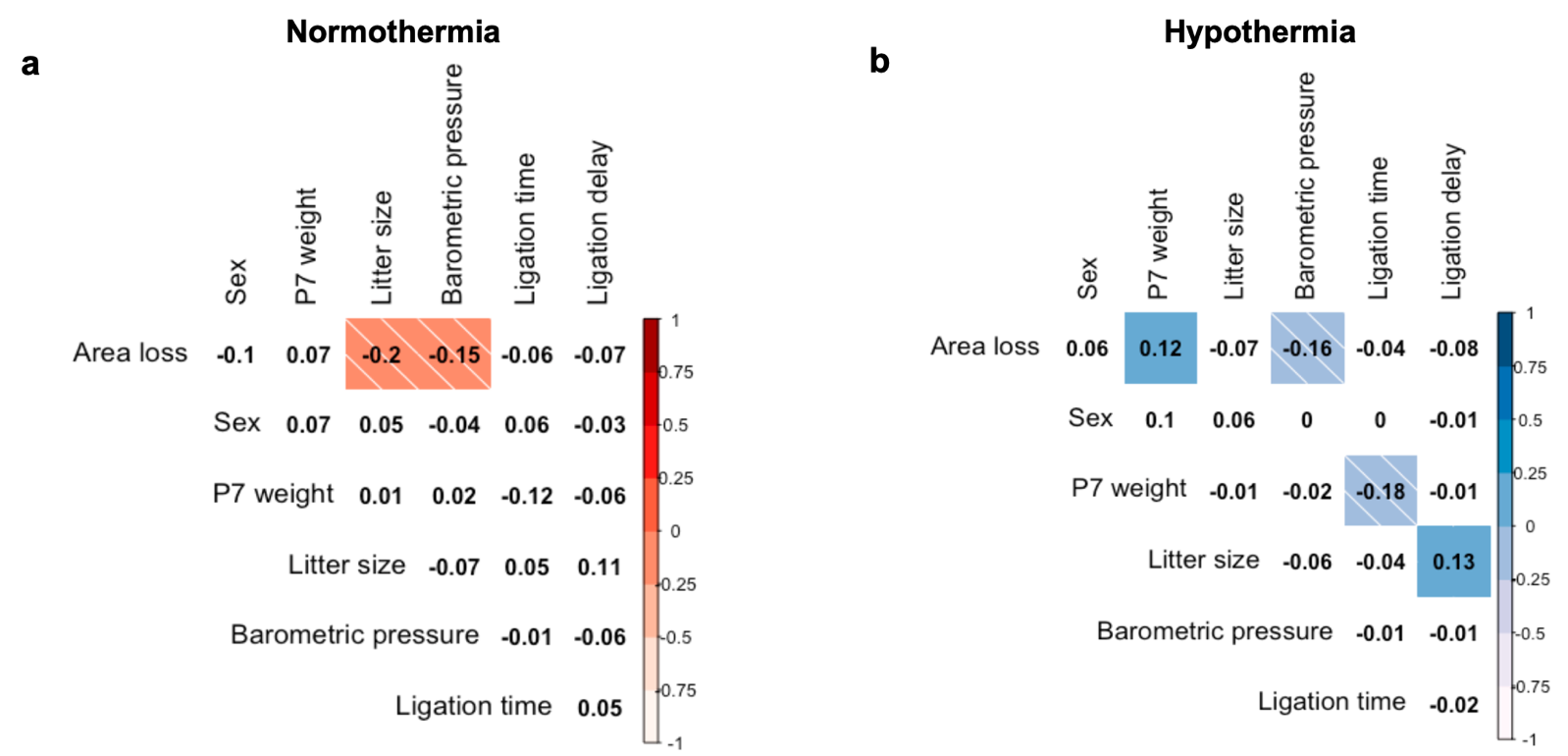


**Supplemental Figure S2.** Spearman correlation plots between experimental parameters and area loss in NT **(A)** and HT (**B**) animals. Associations that are not significant (unadjusted p>0.05) are displayed with white backgrounds. Depth of colour of the boxes denotes strength of association, with Spearman’s r provided in each box. Hatching denotes a negative correlation. In the NT group, barometric pressure during HI and litter size displayed a small but significant association with area loss. In the HT group, P7 weight and barometric pressure displayed small but significant associations with area loss.


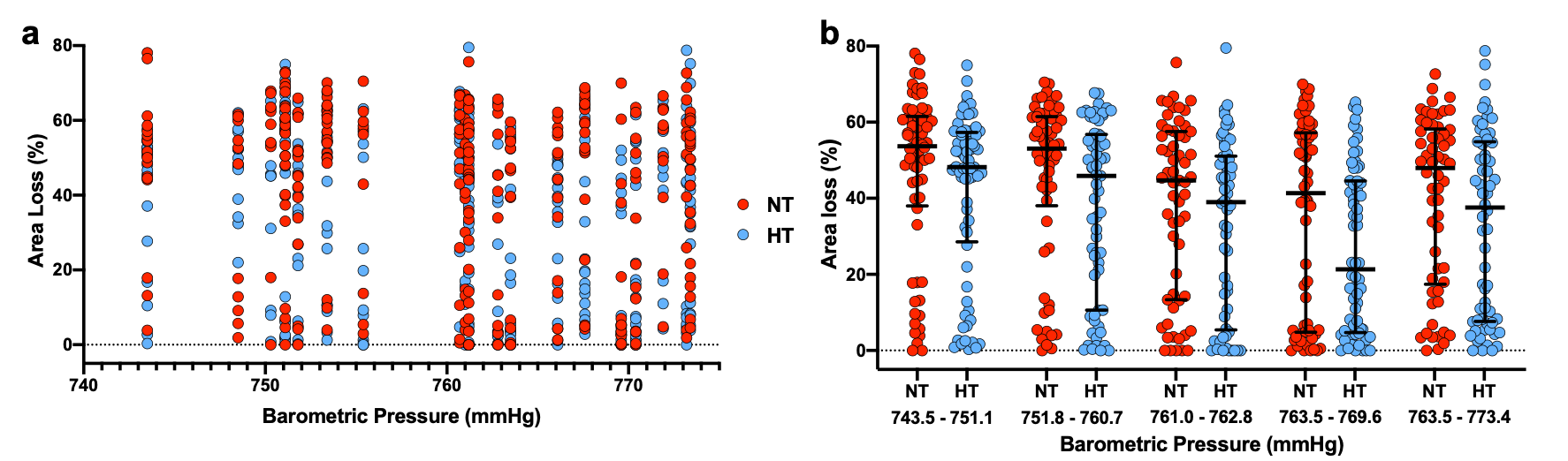

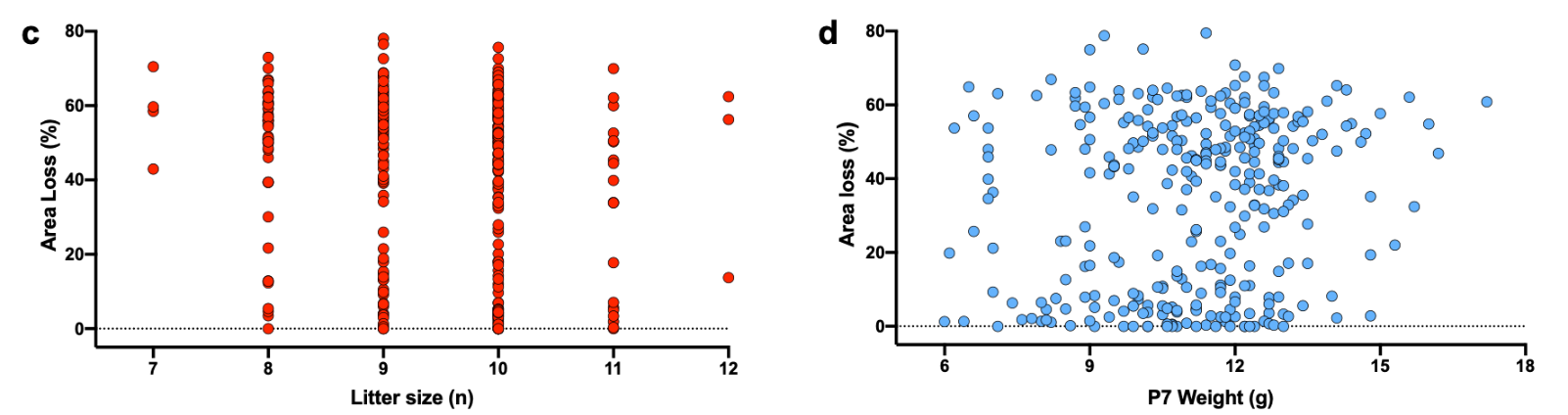

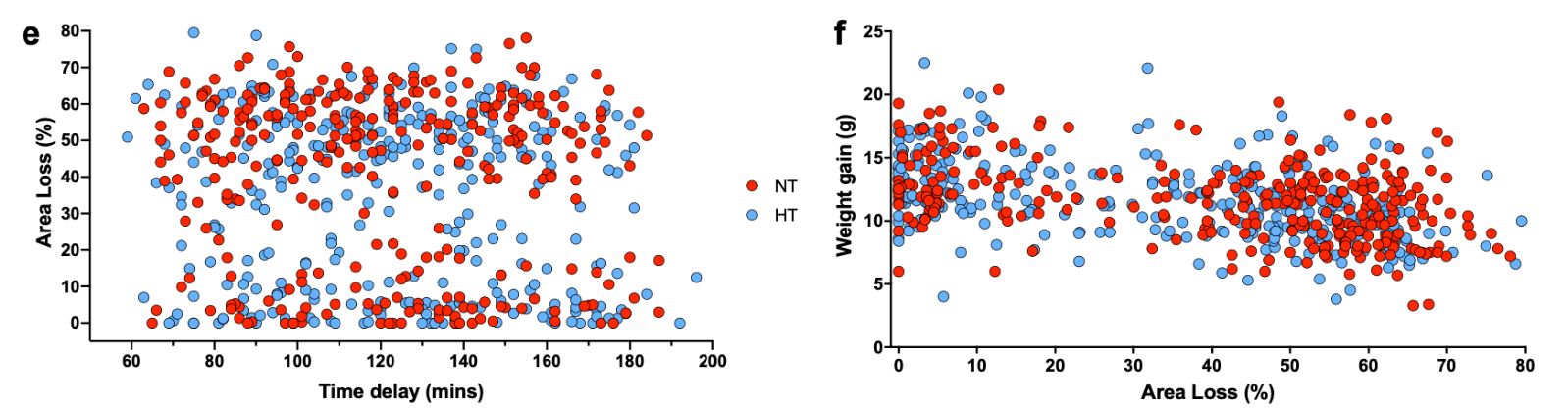


**Supplemental Figure S3.** Barometric pressure versus area loss **(A)** in the NT and HT groups, and plotted by quintiles (n=58-68 per group) of intra-HI pressure **(B)**. Barometric pressure was inversely associated with area loss in both the NT and HT groups (Kendall’s τb, NT p=0.008, HT p=0.005), with an area loss nadir in the 4^th^ quartile (763.5-769.6 mmHg) in both groups. In the NT group litter size **(C)** was negatively correlated with area loss (Kendall’s τb, p<0.001). In the HT group P7 weight **(D)** was positively correlated with area loss (Kendall’s τb, p=0.03). Time delay between ligation and hypoxia **(E)** did not significantly affect area loss in either group (Kendall’s τb, p=0.18 for both). In both the NT and HT groups, area loss was inversely correlated with weight gain **(F)** between P7 and P14 (Kendall’s τb, p<0.01 for both).

**Supplemental Figure S4.** Plot of median (IQR) rectal temperatures during the 5h treatment period across all experiments in both the NT and HT groups.

**Supplemental Table S1. Animal characteristics.** Animal source, number, weight, weight gain, and sex characteristics in each experiment. Weight data is presented as mean (SD). No correlation was seen between experimental outcome (percent neuroprotection in HT group) and animal source, delivery age, weight at P7, weight at P14, or weight gain.

|  |  |  |  |  |  | |  | NT | | HT | | Total | |
| --- | --- | --- | --- | --- | --- | --- | --- | --- | --- | --- | --- | --- | --- |
| Exp. | Breeder | Delivery | No. of Litters | Weight P7 (g) | Weight P14 (g) | Weight gain (g) | | N | % female | N | % female | N | % female |
| 1 | Charles River | P6 | 4 | 11,6 (1,0) | 23,7 (4,0) | | 12,1 (3,9) | 16 | 56 % | 14 | 50 % | 30 | 53 % |
| 2 | Charles River | P6 | 5 | 15,6 (1,3) | 20,6 (2,9) | | 10,0 (2,2) | 14 | 43 % | 14 | 50 % | 28 | 46 % |
| 3 | UiO | N/A | 3 | 14,0 (1,5) | 24,9 (3,7) | | 10,8 (3,1) | 11 | 55 % | 13 | 46 % | 24 | 50 % |
| 4 | Charles River | P6 | 5 | 12,0 (0,9) | 27,9 (2,2) | | 25,9 (1,9) | 15 | 40 % | 15 | 33 % | 30 | 37 % |
| 5 | Charles River | P6 | 5 | 10,0 (0,9) | 22,4 (3,6) | | 12,4 (2,9) | 11 | 73 % | 13 | 62 % | 24 | 67 % |
| 6 | Charles River | P6 | 5 | 11,1 (1,2) | 22,1 (2,9) | | 11,1 (2,2) | 13 | 46 % | 14 | 57 % | 27 | 52 % |
| 7 | Charles River | P6 | 5 | 11,6 (1,2) | 21,2 (2,7) | | 9,6 (2,0) | 10 | 40 % | 10 | 40 % | 20 | 40 % |
| 8 | Charles River | P6 | 5 | 12,9 (1,2) | 23,4 (2,9) | | 10,6 (2,7) | 10 | 50 % | 10 | 50 % | 20 | 50 % |
| 9 | Charles River | P6 | 5 | 11,5 (1,4) | 24,0 (2,8) | | 12,6 (2,0) | 12 | 58 % | 14 | 64 % | 26 | 62 % |
| 10 | Charles River | P6 | 6 | 13,1 (2,0) | 25,6 (4,1) | | 12,6 (2,6) | 18 | 50 % | 18 | 56 % | 36 | 53 % |
| 11 | Charles River | P6 | 5 | 12,2 (1,1) | 22,3 (4,1) | | 10,0 (3,9) | 12 | 42 % | 12 | 50 % | 24 | 46 % |
| 12 | Charles River | P6 | 7 | 11,8 (0,8) | 22,0 (2,4) | | 10,2 (2,0) | 15 | 40 % | 17 | 53 % | 32 | 47 % |
| 13 | Charles River | P6 | 7 | 12,2 (1,2) | 21,7 (2,1) | | 9,6 (1,9) | 12 | 75 % | 12 | 50 % | 24 | 63 % |
| 14 | Charles River | E15 | 7 | 7,9 (1,9) | 18,3 (3,8) | | 10,2 (2,4) | 10 | 70 % | 12 | 75 % | 22 | 73 % |
| 15 | Charles River | E14 | 4 | 8,2 (1,8) | 18,4 (2,9) | | 10,2 (1,8) | 17 | 35 % | 18 | 50 % | 35 | 43 % |
| 16 | Charles River | E14 | 5 | 9,1 (0,9) | 20,8 (2,0) | | 11,7 (2,0) | 15 | 60 % | 17 | 65 % | 32 | 63 % |
| 17 | Charles River | E14 | 4 | 9,1 (1,2) | 19,2 (2,4) | | 10,1 (2,4) | 16 | 50 % | 17 | 35 % | 33 | 42 % |
| 18 | Taconic | P6 | 5 | 11,7 (1,5) | 23,2 (3,8) | | 11,4 (3,8) | 20 | 50 % | 22 | 50 % | 42 | 50 % |
| 19 | Charles River | P6 | 5 | 11,4 (1,0) | 26,3 (3,6) | | 14,8 (3,2) | 22 | 50 % | 19 | 53 % | 43 | 52 % |
| 20 | Charles River | P6 | 4 | 12,2 (0,8) | 22,8 (2,7) | | 10,6 (2,4) | 17 | 59 % | 18 | 72 % | 35 | 66 % |
| 21 | Charles River | P6 | 5 | 11,2 (1,3) | 23,9 (2,9) | | 12,7 (2,0) | 19 | 63 % | 18 | 61 % | 37 | 62 % |
|  | **Overall** |  | **106** | **11,2 (2,0)** | **22,7 (4,0)** | | **11,5 (3,1)** | **305** | **52 %** | **317** | **54 %** | **622** | **53 %** |

Animals were delivered to our institution at postnatal-day 6 (P6), or day 14/15 of pregnancy (E14/E15). UiO = University of Oslo.

**Supplemental Table S2.** Logistic regression model parameter estimates predicting animals in the NT group with injury above the median of all experiments (50.1% area loss).

| **Variable** | **Estimate** | **95% CI** | **p-value** | **Model accuracy** |
| --- | --- | --- | --- | --- |
| Intercept | 29.93 | 9.544 to 50.83 |  | 60.3% |
| Litter size | -0.4161 | -0.6835 to -0.1590 | 0.002 |  |
| Barometric pressure | -0.03419 | -0.06104 to -0.007883 | 0.01 |  |
